# Inferior Gluteal Nerve Originating From the Common Fibular Nerve: A Histologically Validated Anatomical Variant With Clinical Implications

**DOI:** 10.7759/cureus.97939

**Published:** 2025-11-27

**Authors:** Jailenne I Quiñones-Rodríguez, María A Portela-Vázquez, Alexandra N Acevedo-Arroyo, Norman Ramírez-Lluch, Mario Loomis, David J Moeller, Sefik Gokaslan, Dennis Wooten

**Affiliations:** 1 Department of Clinical Anatomy, Sam Houston State University College of Osteopathic Medicine, Conroe, USA; 2 Department of Surgery, School of Medicine University of Puerto Rico, Medical Sciences Campus, San Juan, PRI; 3 Department of Obstetrics and Gynecology, School of Medicine University of Puerto Rico, Medical Sciences Campus, San Juan, PRI; 4 Department of Pediatric Orthopedic Surgery, Mayagüez Medical Center, Mayagüez, PRI

**Keywords:** anatomical variations, gluteus maximus, inferior gluteal nerve, piriformis muscle, sciatic nerve

## Abstract

Detailed knowledge of gluteal region neuroanatomy is essential for accurate clinical diagnosis and for minimizing iatrogenic nerve injury during surgical procedures. Although variations in the origin and course of the inferior gluteal nerve are uncommon, they hold significant clinical importance. During routine cadaveric dissection of the gluteal region, we identified an atypical variation in the origin of the inferior gluteal nerve. In this donor, a unilateral variation was observed in which the inferior gluteal nerve originated from an early bifurcation of the sciatic nerve, emerging from the common fibular nerve. The nerve subsequently passed inferior to the piriformis muscle, coursed medial to the quadratus femoris muscle, and gave rise to multiple motor branches. This anomalous trajectory of the inferior gluteal nerve may increase the risk of nerve injury during commonly performed interventions such as total hip arthroplasty, gluteal intramuscular injections, and flap closure for ischial pressure ulcers. Moreover, such variations may complicate radiologic interpretation and clinical evaluation of gluteal pain syndromes or sciatic neuropathies. Awareness of potential anatomical anomalies in this region is therefore imperative for surgeons, radiologists, and neurologists. Recognition of these variants enhances surgical planning, improves diagnostic accuracy, and reduces the risk of iatrogenic complications. This case highlights the importance of integrating detailed anatomical knowledge into both clinical and surgical practice.

## Introduction

The gluteal region is an important anatomical and clinical landmark that contains muscles and vital neurovascular bundles. Typically, the inferior gluteal nerve (IGN) emerges from the ventral rami of the L5, S1, and S2 spinal nerves and exits the pelvis via the greater sciatic foramen inferior to the piriformis muscle [1-9]. It then divides into multiple branches to innervate the gluteus maximus muscle. However, variations in the origin of the IGN have been documented, presenting anatomical challenges and potential clinical implications [6,8-14]. Thus, awareness of potential variations in the origin and course of the IGN is essential to minimize the risk of iatrogenic injury and preserve the function of the gluteus maximus muscle. Anatomical variations can include differences in the number of branches, the point of origin, or the nerve's course [6,8-14]. The sciatic nerve originates from the ventral rami of the L4, L5, S1, S2, and S3 spinal nerves [3]. It terminates as the tibial nerve (derived from L4-S3) and the common fibular nerve (derived from L4-S2) [3]. It exits the pelvis through the greater sciatic foramen, most commonly entering the gluteal region inferior to the piriformis muscle, a critical anatomical landmark; however, documented anatomical variants demonstrate that components of the sciatic nerve may also pass through or superior to the piriformis muscle [3].

Usually, both components of the sciatic nerve divide at the superior angle of the popliteal fossa, where they supply sensory and motor innervation to the lower limb. However, several variations related to the sciatic nerve division have been described [2-8]. Conversely, the posterior femoral cutaneous nerve is a sensory branch that arises from the ventral rami of the S1 and S2 spinal nerves, and from the ventral rami of the S2 and S3 spinal nerves. It exits the pelvis via the greater sciatic foramen inferior to the piriformis muscle to supply the skin in the posterior thigh, leg, buttock, and perineum. The piriformis muscle is an important anatomical landmark for identifying these structures, as it divides the gluteal region into inferior and superior quadrants.

Awareness of nerve injury when performing procedures in the deep gluteal region, particularly during total hip arthroplasty performed through a posterior approach or other operations requiring exposure of the short external rotators. Although clinically evident peripheral nerve palsy following primary total hip arthroplasty is relatively uncommon, with reported incidences of sciatic nerve palsy of approximately 0.6-3.8% and overall nerve injury rates ranging from about 0.1-3.7% in primary cases and up to 7.6% in revision procedures [15-17], its consequences can be profound. Regional anatomical studies have demonstrated substantial variation in the course and division patterns of the sciatic nerve and its relationship to the piriformis muscle, an area frequently encountered during posterior hip exposure. Similar variability in adjacent structures, including the IGN, may further influence surgical visualization and elevate the risk of iatrogenic injury during gluteal procedures [3,8,15,16].

Injury to the IGN can result in weakness or atrophy of the gluteus maximus muscle, leading to impaired hip joint extension and a characteristic lurching gait [4]. Given the potential for functional compromise and the anatomical variability within the deep gluteal region, careful consideration of nerve course and branching patterns is essential during both diagnostic evaluation and surgical intervention. Within this context, the present case describes an uncommon anatomical variation identified in an elderly female donor, characterized by the IGN arising from the common fibular nerve after an early bifurcation of the sciatic nerve at the pelvis. This configuration underscores the clinical importance of recognizing variant anatomy that may influence surgical exposure, nerve preservation, and procedural safety in the gluteal and posterior hip joint region.

## Case presentation

During a routine cadaveric dissection of an elderly female, a unilateral anatomical variation of the IGN and sciatic nerve was identified. The clinical and family history, as well as the cause of death, were unavailable.

Dissection methodology

A bilateral dissection of the gluteal region was performed using a lateral-to-medial approach. Initially, skin incisions were made, removing superficial and deep fasciae and revealing the gluteus maximus muscle. A lateral incision of the gluteus maximus muscle was performed, and muscle flaps were reflected to expose the underlying structures.

Right Side Dissection Findings

On the right side, the dissection proceeded without any anatomical variations. The gluteus medius muscle and the piriformis muscle were revealed. At the superior border of the piriformis muscle, the superior gluteal neurovasculature was identified. The inferior gluteal neurovasculature was observed at the inferior border of the piriformis muscle. The origin and distribution of all other anatomical structures on the right side were typical.

Left Side Dissection Findings

**Figure 1:**
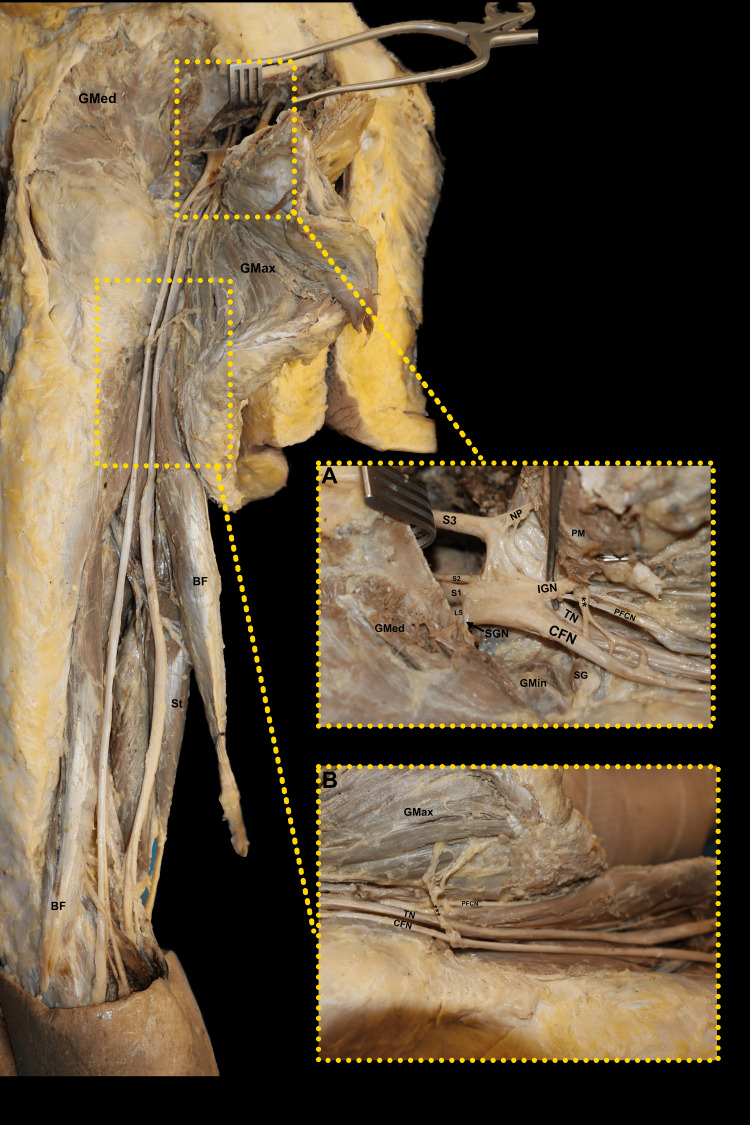
Anatomical variation in the left gluteal region showing the inferior gluteal nerve (IGN) originating from the common fibular nerve (CFN) after early sciatic nerve (SN) bifurcation and supplying the gluteus maximus muscle (Gmax) (A) A magnified view illustrates the early bifurcation of the sciatic nerve (SN) into the common fibular nerve (CFN), originating from spinal nerves L4-S2, and the tibial nerve (TN), originating from L4-S3. The inferior gluteal nerve (IGN) is shown arising from the CFN and contributing to the innervation of the gluteus maximus muscle (GMax). Also visible are the superior gluteal nerve (SGN), which innervates the gluteus medius muscle (GMed) and gluteus minimus muscle (GMin), and the nerve to the piriformis (NP) coursing toward the piriformis muscle (PM). The posterior femoral cutaneous nerve (PFCN) follows its typical anatomical course without variation. The superior gemellus (SG) muscle is also visible in relation to the exiting nerve branches. (B) The early bifurcation of the CFN and TN continues along their typical trajectories toward the popliteal fossa, where they supply the lower limb. The CFN gives off branches contributing to the formation of the IGN (**), which enters the GMax, and extends toward the posterior compartment of the thigh (***). Muscles of the posterior thigh, including the semitendinosus (ST) and biceps femoris (BF), are seen in proximity to these neurovascular structures.

On the left side, a significant anatomical variation was identified during dissection. After reflecting the gluteus maximus muscle, it was noted that the sciatic nerve bifurcated earlier than usual, separating into the common fibular nerve laterally and the tibial nerve medially. This bifurcation occurred inferior to the piriformis muscle, with both branches emerging through the gluteal region and continuing distally along the posterior aspect of the thigh. The tibial nerve and the common fibular nerve remained distinct throughout their course, continuing separately toward the popliteal fossa along their usual anatomical distributions. A meticulous dissection of the sacral plexus toward the midline further corroborated the presence of this early bifurcation pattern, confirming its deviation from the conventional anatomy (Figure 1).

The variation highlights the dual contribution of the IGN to the gluteus maximus muscle, both with its normal course running with the inferior gluteal vascular bundle, and its branches originating from the common fibular nerve. The IGN receives motor input from fibers derived from the same L4, L5, S1, and S2 spinal nerve contributions, ensuring innervation to the gluteus maximus muscle (Figure 1A). This anatomical finding demonstrates a complex nerve branching pattern that reinforces the gluteal muscle innervation, with additional branches observed arising medial to the quadratus femoris muscle (Figure 1B).

The common fibular nerve is formed by fibers originating from the ventral rami of the L4-S2 spinal nerves, while the tibial nerve arises from the ventral rami of the L4-S3 spinal nerves (Figure 1). This early bifurcation occurs proximal to the typical location, within the gluteal region, instead of the distal thigh. The posterior femoral cutaneous nerve, arising from the sacral plexus independently of the common fibular nerve and tibial nerve, is also visualized without anatomical variation, maintaining its typical trajectory through the posterior thigh (Figure 1A).

Tissue processing and sectioning

**Figure 2:**
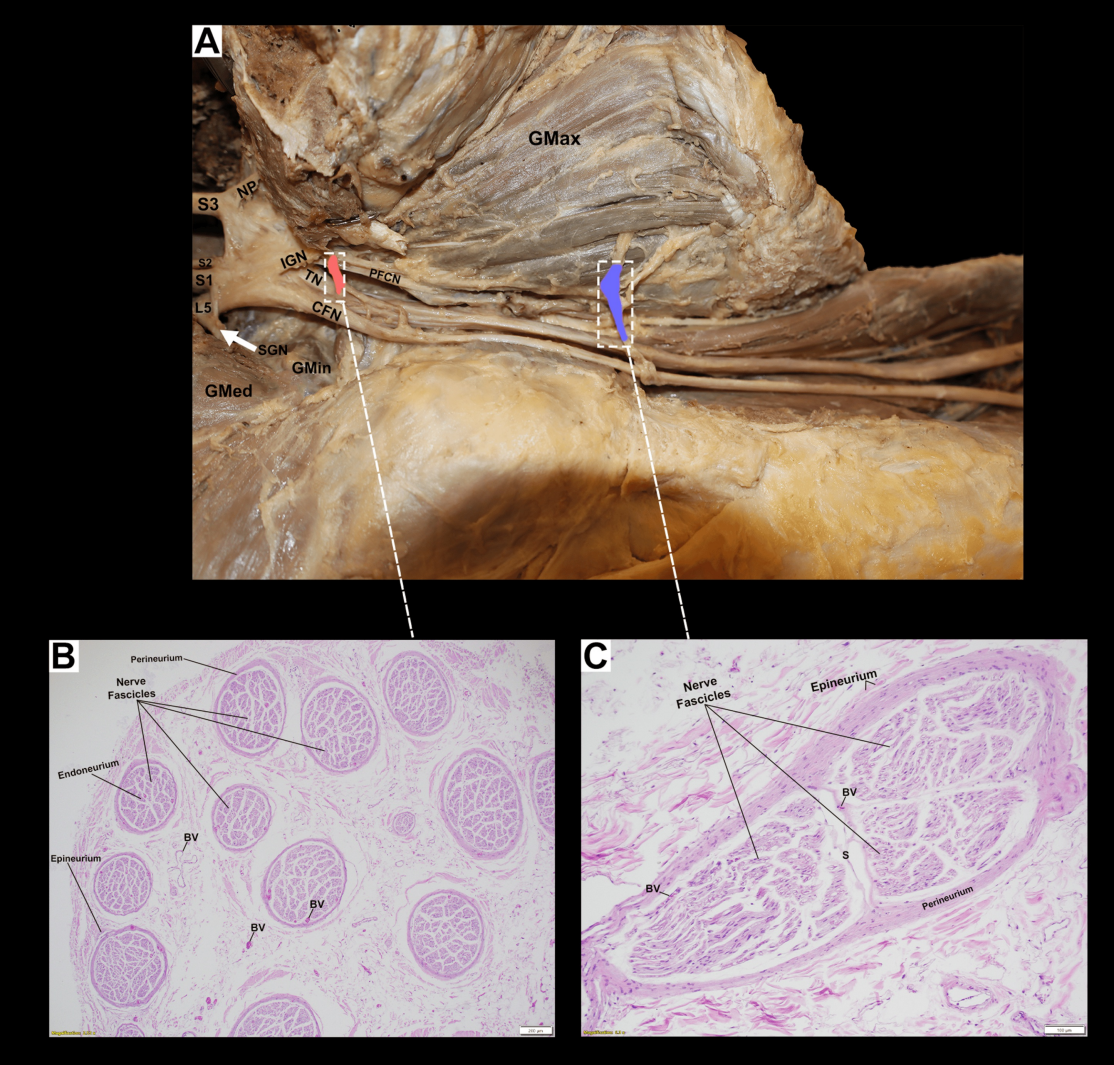
Gross anatomical depiction of the variant inferior gluteal nerve origin with accompanying histological confirmation of neural tissue (A) Cadaveric dissection illustrating an early bifurcation of the sciatic nerve (SN) into the tibial nerve (TN), originating from L4-S3, and the common fibular nerve (CFN), originating from L4-S2. The IGN is shown branching anomalously from the CFN and innervating the gluteus maximus muscle (GMax). Two additional nerve branches, highlighted in coral and purple, are observed emerging from the CFN and entering the GMax. The gluteus medius muscle (GMed), gluteus minimus muscle (GMin), piriformis muscle (PM), superior gluteal nerve (SGN), nerve to the piriformis (NP), posterior femoral cutaneous nerve (PFCN), and superior gemellus muscle (SG) are also labeled. The PFCN follows its typical anatomical trajectory without variations. (B) Histological section (H&E stain) of the coral-identified branch, dissected from the CFN, confirms the presence of nervous tissue. (C) Histological section (H&E stain) of the purple-identified branch, dissected from the CFN, also confirms the presence of nervous tissue. BV, blood vessels.

The excised nerve samples were obtained from the peripheral branches contributing to the distribution of the IGN into the gluteus maximus muscle (Figure 2A). Two distinct samples were collected to confirm neural identity and differentiate from connective tissue: (i) fibers interconnecting the common fibular nerve and IGN, and (ii) fibers originating directly from the common fibular nerve and innervating the gluteus maximus muscle.

Following excision, samples were immediately fixed in 10% neutral-buffered formalin (NBF) for 24 hours. The tissues were then processed using the Sakura Tissue-Tek VIP 5 Tissue Processor (Sakura Finetek, Torrance, CA), which included sequential dehydration in graded ethanol, clearing in xylene, and infiltration with paraffin. Serial sections of 5-7 µm thickness were obtained using a rotary microtome and mounted on glass slides.

Hematoxylin and Eosin (H&E) Staining

Paraffin sections were deparaffinized in xylene, rehydrated through a graded ethanol series, and rinsed in distilled water. Hematoxylin and eosin staining was performed using the StatLab H&E Staining Kit (StatLab, McKinney, TX), following the manufacturer’s protocol. Hematoxylin staining was used to highlight nuclear structures, followed by eosin counterstaining for cytoplasmic and extracellular matrix visualization. The stained sections were then dehydrated, cleared, and coverslipped using a permanent mounting medium for histological evaluation under light microscopy.

Histology Findings

Histological evaluation demonstrated morphological features consistent with peripheral nerve tissue. The examined sections (Figures 2B-2C) showed multiple well-organized nerve fascicles surrounded by a distinct perineurial layer, with interposed endoneurial connective tissue. Within these fascicles, numerous eosinophilic axonal profiles were observed, accompanied by elongated basophilic nuclei characteristic of Schwann cells. Small intrafascicular and perineurial blood vessels were also present, further supporting the identification of the sampled structure as a peripheral nerve.

The neuroanatomical features consistently seen in both the coral- and purple-identified branches confirm that these are actual nervous tissues (Figures 2B-2C). These histological findings provide objective support for the gross anatomical observation of variant branches arising from the common fibular nerve and contributing to the regional innervation pattern.

## Discussion

IGN variations frequency and distribution

Anatomical variations of the IGN can present challenges during surgical and interventional procedures in the gluteal region [8-11]. Although a few studies have described its relationship to the piriformis muscle, the available evidence indicates that atypical IGN patterns are uncommon and likely underreported. Reported deviations include a high sciatic division in which an anomalous double root emerged from both the upper and lower borders of the piriformis muscle to form the IGN [5], as well as instances in which the nerve originated from the sciatic trunk without additional structural deviations [12].

Systematic reviews and extensive anatomical studies show that variations in the sciatic nerve, particularly in its level of division and its relationship to the piriformis muscle, are relatively common, with pooled estimates indicating that approximately 16-17% of individuals exhibit a departure from the typical single-trunk configuration [18-26]. These variations typically involve a high division or an altered course of one of the sciatic components as it exits the pelvis. In contrast, deviations involving the IGN itself are far less frequently documented. Isolated descriptions note its emergence superior to the piriformis muscle in specific populations [9], and rare reports describe communicating branches between the posterior femoral cutaneous nerve and the IGN [10]. The variant identified in the present case, an IGN arising from the common fibular division following an early sciatic bifurcation, differs from these patterns, is not represented in pooled anatomical analyses, and has been described only rarely in individual case reports [13,14].

Clinical significance

Surgical Considerations: Orthopedic Surgery 

Injury to the IGN is most often iatrogenic, typically occurring in the setting of pelvic pathology, intramuscular injections, or surgical exposure within the deep gluteal region. Among operative procedures, total hip arthroplasty performed through a posterior approach carries particular risk due to the nerve's close relationship to the deep surface of the gluteus maximus muscle and its course across the operative field [27]. The Kocher-Langenbeck posterior approach is widely utilized because it provides excellent visualization of the acetabulum and proximal femur, facilitates accurate component positioning, and avoids extensive dissection of the abductor muscles of the thigh, often resulting in shorter operative times and more efficient access for complex reconstructions [28-30]. Despite these advantages, the proximity of the IGN places it at risk during muscle splitting, retraction, or instrumentation along the posterior hip corridor. Although the precise incidence of IGN injury remains unknown, peripheral nerve injuries during total hip arthroplasty overall have been reported to range from approximately 0.5% to 8%, depending on patient factors and surgical approach [31].

Beyond iatrogenic injury, the IGN may also be affected in conditions such as piriformis syndrome or during peri-sacral surgical procedures [32]. Although uncommon, IGN entrapment can contribute to deep buttock or posterior pelvic pain and is often misattributed to myofascial sources, delaying appropriate diagnosis [33]. Loss of the IGN function produces weakness of the gluteus maximus muscle, resulting in impaired hip joint extension and characteristic gait disturbance. The critical complications of IGN injury include alteration of the gait cycle, a gluteus maximus “lurch,” and ipsilateral muscle wasting that leads to loss of the normal buttock contour, collectively contributing to reduced functional capacity and quality of life. These clinical consequences underscore the importance of identifying anatomical variations of the IGN, as atypical origins or courses may influence susceptibility to compression, misdiagnosis, or surgical injury.

The variant branches arising from the common fibular nerve and coursing with the IGN are best interpreted as additional motor branches supplying the gluteus maximus muscle, rather than somatosensory fibers. The IGN is predominantly a motor nerve, and cutaneous sensation in the buttock is normally mediated by the inferior cluneal branches of the posterior femoral cutaneous nerve. Therefore, injury to this anatomical variant would be expected to produce motor deficits, such as impaired hip joint extension, gait disturbance, and gluteus maximus atrophy, rather than primary sensory loss. Recognizing such putative motor branches originating atypically from the common fibular nerve is clinically relevant, as their unusual origin and trajectory may influence vulnerability to iatrogenic injury during surgical exposure or complicate intraoperative nerve identification.

Radiological imaging, such as magnetic resonance neurography, can assist in identifying anatomical variations; however, the small caliber of the IGN may limit its visibility. Complementary diagnostic tools, including electromyography, nerve conduction studies, intraoperative nerve stimulation, and careful use of anatomical landmarks, enhance the ability to identify the IGN and its variants during clinical assessment and surgical procedures, thereby reducing the risk of nerve injury.

Surgical Considerations: Plastic and Reconstructive Surgery

The anatomical variation identified in this case has important implications for reconstructive procedures addressing ischial pressure sores, particularly in ambulatory patients who depend on preserved gluteus maximus muscle function for gait stability. In standard posterior thigh-based flap reconstruction, dissection is performed along the deep (undersurface) plane of the gluteus maximus muscle near its inferior border and over the hamstring origin. Although the sciatic nerve typically lies deep and lateral to this operative field, the variant IGN observed here, arising from an early division of the common fibular nerve and ascending toward the gluteus maximus, crosses directly within this deep fascial plane beneath the muscle. A motor branch positioned in this location is atypical and would be susceptible to transection during inferior gluteus maximus mobilization. Based on this single anatomical observation, a cautious dissection zone may include maintaining awareness of potential neural structures coursing immediately superficial to the short external rotators and lateral to the ischial tuberosity when elevating the inferior aspect of the gluteus maximus. Injury to such a branch could compromise hip-extension strength and contribute to gait disturbances in ambulatory patients.

## Conclusions

This case documents an uncommon anatomical variation in which the IGN arose from the common fibular division of the sciatic nerve following an early bifurcation. The variant motor branch ascended through the deep plane beneath the gluteus maximus muscle, placing it directly within the deep gluteal surgical plane, frequently used during posterior hip exposure and reconstructive procedures for ischial pressure sores. Awareness of this atypical trajectory is essential, as unrecognized injury to the IGN can compromise gluteus maximus muscle function and contribute to gait disturbances in ambulatory patients. Recognition of such variations may also facilitate more accurate intraoperative nerve identification and support safer surgical planning in the deep gluteal region. Although imaging modalities such as magnetic resonance neurography have limited sensitivity for small-caliber motor nerves, preoperative consideration of potential variant pathways and vigilant dissection can help reduce the risk of iatrogenic injury.

Overall, this case emphasizes the importance of understanding potential variations in the origin and course of the IGN, particularly when operating in regions where the gluteus maximus muscle is mobilized or the deep gluteal interval is exposed. The accompanying histological confirmation reinforces the neural identity of the variant branch and supports the anatomical relevance of this finding.
